# Neurophysiological approaches for managing pain in multiple sclerosis: a mini review

**DOI:** 10.3389/fnhum.2025.1552435

**Published:** 2025-02-28

**Authors:** Samar S. Ayache, Moussa A. Chalah

**Affiliations:** ^1^Department of Neurology, Gilbert and Rose-Marie Chagoury School of Medicine, Lebanese American University, Byblos, Lebanon; ^2^Institut de la Colonne Vertébrale et des NeuroSciences (ICVNS), Centre Médico-Chirurgical Bizet, Paris, France; ^3^EA4391 Excitabilité Nerveuse and Thérapeutique, Université Paris Est Créteil, Créteil, France; ^4^Department of Clinical Neurophysiology, DMU FIxIT, Henri Mondor University Hospital, Assistance Publique-Hôpitaux de Paris (APHP), Créteil, France; ^5^Institut de Neuromodulation, Service Hospitalo-Universitaire, Pôle Hospitalo-Universitaire Psychiatrie Paris 15, Hôpital Sainte-Anne, GHU Paris Psychiatrie et Neurosciences, Paris, France

**Keywords:** multiple sclerosis, pain, noninvasive brain stimulation, transcranial direct current stimulation, tDCS, transcranial magnetic stimulation, symptom cluster

## Abstract

Chronic pain is a prevalent yet often under-recognized symptom among individuals with multiple sclerosis (MS), affecting 29–86% of the population. This condition can significantly impact the individuals' functionality, including their capacity to engage in professional activities. The pathophysiology underlying this condition remains intricate and not fully elucidated, and inadequate responses to pharmacological interventions or adverse effects can hinder its management. In light of these observations, there is an urgent need to identify new therapeutic interventions. Non-invasive brain stimulation (NIBS) techniques hold promise for addressing MS-related pain. This mini-review aims to analyze the findings from studies using NIBS techniques, such as transcranial direct current stimulation (tDCS) and repetitive transcranial magnetic stimulation (rTMS), to assess their analgesic potential in people with MS. Seven relevant reports are available. Five of these studies used tDCS, one utilized a transcranial random noise stimulation (tDCS variant), and one compared rTMS with transcranial theta burst stimulation (rTMS variant). The results indicate the potential benefits of NIBS for pain management in MS. However, the study's limitations, including the scarcity of data, small sample size, the limited number of sessions, sham design, and brief follow-up, are also noted and discussed. Finally, directions for future research are suggested.

## 1 Introduction

Multiple sclerosis (MS) is an autoimmune disease of the central nervous system that involves demyelination, neurodegeneration, and synaptopathy at the level of the brain and spinal cord. People with MS (PwMS) experience a wide range of manifestations, including overt symptoms such as motor deficits, balance and coordination disorders, and speech abnormalities, as well as invisible symptoms that are garnering increasing attention. The latter, while not overtly visible to others, can be debilitating and distressing symptoms, potentially acting as significant predictors of health distress. Those symptoms may include fatigue, emotional manifestations, cognitive deficits, and pain (White et al., 2008).

Chronic pain, a prevalent yet under-recognized symptom, affects a significant proportion of PwMS, ranging from 29 to 86% of the population (O'Connor et al., 2008). It is reported by 12% of PwMS as the worst symptom (Kenner et al., 2007). The symptoms can occur in several types, including trigeminal neuralgia, headaches, back pain, Lhermitte's sign, painful tonic spasms, and extremity pain (O'Connor et al., 2008; McBenedict et al., 2024). Among these, lower limb dysesthesia, characterized by a continuous burning sensation with exacerbation during the nocturnal period and with physical activity, appears to be the most common and difficult-to-manage type. The mechanisms underlying this type of pain in MS remain to be fully elucidated. One hypothesis suggests that lower limb dysesthesia may stem from pathophysiological mechanisms involving the central sensory pathways (nociceptive spinothalamic tracts), encompassing inflammation, demyelination, and axonal loss within these pathways. Other proposed mechanisms include central sensitization, dysfunction of GABAergic interneurons responsible for “cold inhibition of pain,” neuronal hyperexcitability secondary to demyelination, and acquired channelopathy (Kenner et al., 2007; O'Connor et al., 2008; Seixas et al., 2014; McBenedict et al., 2024). Furthermore, the findings from the existing neuroimaging data, albeit limited in scope, suggest an association between pain in MS and lesions mainly affecting the brainstem and spinal cord, as well as the thalami and several levels of the pyramidal tract (Seixas et al., 2014). An association has been identified between MS pain and some sociodemographic and clinical variables, namely age, disease duration, the presence of other invisible symptoms, and the level of functional impairment (O'Connor et al., 2008).

Pain can drastically impact individuals' functioning, including their ability to work (O'Connor et al., 2008). The pathophysiology of pain in MS remains intricate and poorly understood, impeding effective management strategies due to limited treatment responses or adverse effects of therapeutic interventions such as muscle relaxants, anticonvulsants, antidepressants, and opioids (Shkodina et al., 2024).

Given the prevalence and impact of pain in MS and the modest efficacy of the available interventions, the identification of new therapeutics appears critical. Therefore, neurophysiological techniques—such as non-invasive brain stimulation (NIBS)—may constitute novel therapeutic strategies. This mini-review aims to analyze the findings of all studies that used NIBS techniques, such as transcranial direct current stimulation (tDCS) and repetitive transcranial magnetic stimulation (rTMS), to assess their potential for providing analgesia in PwMS. The limitations of the extant literature are addressed, and recommendations for future research are provided.

## 2 Research method

A comprehensive search was conducted by both authors in PubMed, Medline, and Scopus to identify original research articles on using NIBS in the context of pain in MS. The search used the following inclusion criteria: articles published in English or French at any time up to October 2024. The following key terms were used: (“MS” OR “multiple sclerosis”) AND (“pain”) AND (“non-invasive brain stimulation” OR “NIBS” OR “transcranial magnetic stimulation” OR “TMS” OR “theta burst stimulation” OR “TBS” OR “transcranial electrical stimulation” OR “tES” OR “transcranial direct current stimulation” OR “tDCS” OR “transcranial random noise stimulation” OR “tRNS”). The bibliographic references of the retrieved articles were also scanned to identify additional relevant references.

## 3 Neurophysiological approaches targeting pain in multiple sclerosis

Over the past decade, there has been increasing interest in assessing the effects of NIBS on commonly encountered symptoms in MS. A growing body of literature has opted for tDCS or its variants rather than rTMS in PwMS (Ayache and Chalah, 2018). tDCS involves applying a weak electrical current (1–4 mA) over the skull for 20 min, targeting brain regions of interest via surface electrodes connected to a battery-driven stimulator. The placement of the anode or the cathode over the cerebral region of interest results in an increase or a decrease in resting membrane potential excitability. The knowledge of tDCS effects on cortical excitability derives from studies applying this technique to the motor cortex, where different parameters led to an increase (in the case of anodal tDCS) or a decrease (in the case of cathodal tDCS) in the amplitude of motor-evoked potentials. Transcranial random noise stimulation (tRNS) is a variant of tDCS that, like the latter, uses a weak electrical current. The difference is that the current oscillates randomly between 0.1 and 640 Hz, following a Gaussian curve around a central point.

rTMS delivers electromagnetic pulses over the skull using a coil connected to a magnetic stimulator. Different stimulation parameters exist, with excitatory or inhibitory effects on cortical excitability depending on whether high-frequency (HF, ≥5 Hz) or low-frequency (LF, ≤ 1 Hz) rTMS is applied, at least in the case of the motor cortex. However, it is important to remember that this vision is simplistic and that the effects are more complex and depend on several variables. Theta burst stimulation (TBS) is a specific rTMS paradigm with a frequency that mimics the endogenous electroencephalographic theta rhythm (5 Hz) and allows the delivery of many pulses in a shorter duration compared to classical rTMS. Similar to HF and LF rTMS, intermittent and continuous TBS (iTBS and cTBS) elicit excitatory and inhibitory effects, respectively. Regarding the mechanisms of action, knowledge of the effects of these techniques stems from neurophysiological studies involving the motor cortex, where HF rTMS/iTBS increases the amplitude of motor-evoked potentials, whereas LF rTMS/cTBS induces the opposite effect.

Irrespective of the disease, European guidelines on the use of these techniques suggest a possible efficacy of anodal tDCS over the primary motor cortex (M1) contralateral to the pain side in individuals with lower limb neuropathic pain, at least when it is due to spinal cord lesions (level C evidence) (Lefaucheur et al., 2020). In addition, a definite efficacy has been suggested for using HF rTMS over M1 contralateral to the painful side in the context of neuropathic pain (Lefaucheur et al., 2020).

In total, seven studies published in the English language have used NIBS techniques for pain relief in MS: five randomized controlled trials (RCTs) applying tDCS (of which one uses tRNS), one case report using tDCS, and one RCT using iTBS and HF rTMS (Table 1).

**Table 1:** Studies applying non-invasive brain stimulation techniques to treat neuropathic pain in people with multiple sclerosis.

**References**	**Study design**	**Population**	**Intervention**	**Outcome**	**Results**
Mori et al. (2010)	RCT, double-blind, sham-controlled, crossover	•11F/8M •19 RR •Active group: 5F/5M •Age: 42.8 ± 11.8 years •EDSS: 2.4 ± 1.7 •DD: 10.1 ± 9.9 years •Sham group: 6F/3M •Age: 46.3 ± 16.1 years •EDSS: 2.4 ± 1.2 •DD: 10.3 ± 7.3 years	•tDCS •Current intensity: 2 mA •Electrode size: 35 cm^2^ •Anode: M1 (C3/C4 contralateral to the pain side) •Cathode: contralateral supraorbital area •Session duration: 20 min •Five consecutive daily sessions per group	Neuropathic pain (Visual Analog Scale, Short Form McGill Pain Questionnaire), anxiety, depression, quality of life	Significant analgesic effects and improvement in quality of life lasting up to Week 4
Ayache et al. (2016)	RCT, double-blind, sham-controlled, crossover	•13F/3M •Age: 48.9 ± 10.0 years •11RR/4SP/1PP •EDSS: 4.25 ± 1.4 •DD: 11.8 ± 9.4 years	•tDCS •Current intensity: 2 mA •Electrode size: 25 cm^2^ •Anode: F3 •Cathode: contralateral supraorbital area (AF8) •Session duration: 20 min •Three consecutive daily sessions	Neuropathic pain (Visual Analog Scale, Brief Pain Inventory), cognition (attention), fatigue, anxiety, depression, neurophysiological measures (pain-related evoked potentials and frontal mid-line θ activity)	Significant analgesic effects
Palm et al. (2016)	RCT, double-blind, sham-controlled, crossover	•13F/3M •Age: 47.4 ± 8.9 years •11RR/4SP/1PP •EDSS: 4.2 ± 1.3 DD: 12.5 ± 9.1 years	•tRNS •Current •intensity: 2 mA •Electrode size: 25 cm^2^ •Anode: F3 •Cathode: contralateral supraorbital area (AF8) •Session duration: 20 min •Three consecutive daily sessions	Neuropathic pain (Visual Analog Scale, Brief Pain Inventory), cognition (attention), fatigue, anxiety, depression, and neurophysiological measures (pain-related evoked potentials and frontal mid-line θ activity)	Tendency toward a decrease in the amplitude of pain-related evoked potentials (N2–P2) and improvement in pain without changes in the remaining parameters
Rudroff et al. (2019)	Case report	•52-year-old man •RR MS •DD: 13 years •Patient-determined disease step score: 3 (moderate disability)	•tDCS •Current intensity: 2 mA •Electrode size: 25 cm^2^ •Anode: M1 (C3, contralateral to the pain side) •Cathode: contralateral supraorbital area •Session duration: 20 min •Five consecutive daily sessions	Neuropathic pain (Visual Analog Scale, Neuropathic Pain Symptom Inventory), [^18^F] fluorodeoxyglucose positron emission tomography	Improvement in pain scores accompanied by a normalization in thalamic metabolism bilaterally
Korzhova et al. (2019)	RCT, double-blind, sham-controlled, parallel	•20F/14M •Age NP •34 SP •EDSS: 6.5 •DD: 4–20 years	•iTBS/rTMS •Target location: M1 •Using figure of eight coil •Intensity: 80% resting motor threshold •rTMS group parameters: 20 Hz, 1,600 pulses/session •iTBS group parameters: 5–35 Hz, 1,200 pulses per session •Five consecutive daily sessions per week for 2 weeks	Spasticity-related pain (a scale of pain levels associated with spasticity), fatigue, and spasticity	•Improvement in pain and fatigue with effects lasting 2 weeks post-intervention in the high-frequency rTMS group •Improvement in spasticity in both treatment groups
Workman et al. (2020)	RCT, double-blind, sham-controlled, crossover pilot study	•3F/3M •Age: 46.7 ± 14.1 years •6RR •Disability: moderately disabled •DD: NP	•tDCS •Current intensity: 2 mA •Electrode size: 35 cm^2^ •Anode: M1 representation of the more affected leg •Cathode: contralateral supraorbital area •Session duration: 20 min •Five consecutive daily sessions	Pain (Visual Analog Scale), performance fatigability, fatigue, and depression	•Significant reduction in pain, fatigability, and fatigue •No significant changes in depression scores
Young et al. (2020)	RCT, single-blinded, sham-controlled, parallel	•24F/6M •Age: active group: 51.2 ± 9.3 years •Sham group: 49.87 ± 12.9 years •16RR/11SP/3PP •EDSS/DD NP	•tDCS •Current intensity: 2 mA •Electrode size: 35 cm^2^ •Anode: M1 (C3/C4 contralateral to the pain side) •Cathode: contralateral supraorbital area •Session duration: 2x10 min separated by 25 min of stimulation-free interval •Five consecutive days	Neuropathic pain (Visual Analog Scale, Short Form McGill Pain Questionnaire, Neuropathic Pain Scale), anxiety, depression, stress, and quality of life	Significant analgesic after-effects that lasted 2 weeks but not 4 weeks later No significant effects on anxiety, depression, stress, or quality of life

DD, disease duration; EDSS, Expanded Disability Status Scale; DD, disease duration; iTBS, intermittent theta burst stimulation; NP, not provided; PP, primary progressive; RR, relapsing remitting; SP, secondary progressive; tDCS, transcranial direct current stimulation; TMS, transcranial magnetic stimulation; and tRNS, transcranial random noise stimulation. Electrode location is based on the 10–20 international classification for electroencephalographic electrode positioning. Quantitative data are primarily presented as mean ± SD when available.

The first RCT applied anodal tDCS over M1 contralateral to the pain side for five consecutive daily sessions (20 min per session) in 19 relapsing-remitting (RR) PwMS suffering from neuropathic pain (Mori et al., 2010). Compared to sham stimulation, anodal tDCS resulted in significant analgesic effects [according to the Visual Analog Scale (VAS) and the Short Form McGill Pain Questionnaire (MPQ)] and an improvement in quality of life without impacting affective symptoms (anxiety or depression). Such an improvement emerged after the third stimulation session and remained significant 3 weeks later. A second sham-controlled RCT used similar stimulation parameters, electrode location, and protocol duration in 30 RR PwMS with neuropathic pain (Young et al., 2020). However, the participants received two 10-min sessions each day, separated by a 25-min stimulation-free interval, in an attempt to induce cumulative neuroplastic effects (Bastani and Jaberzadeh, 2014). Here, five consecutive daily sessions of anodal tDCS resulted in significant analgesic effects (VAS and short-form MPQ) that remained significant at 2 weeks but not at 4 weeks, with no changes in the remaining outcomes, including anxiety, depression, stress, and quality of life. Splitting the daily session, as seen in the study by Young et al., did not help improve the efficacy of analgesia, which did not last more than 2 weeks, unlike the results obtained by Mori et al., when applying the 20-min sessions. In the third study (case report), five consecutive daily sessions of anodal tDCS were applied over M1 contralateral to the neuropathic pain site in one individual with RR MS (20 min per session). The participant underwent clinical evaluation and [^18^F] fluorodeoxyglucose positron emission tomography ([^18^F] FDG-PET), which aims to assess the mechanisms of action of tDCS in this context (Rudroff et al., 2019). Interestingly, the tDCS-induced analgesic effects (according to the VAS and Neuropathic Pain Symptom Inventory) were accompanied by a normalization of the thalamic hypometabolism found before the intervention. Such a finding is consistent with the thalamic incrimination in the pathophysiology and modulation of pain. Here, it is worth noting that studies suggest that tDCS over M1 does not target the motor system but rather horizontal fibers that pass through the precentral gyrus and are involved in pain modulation (Nguyen et al., 2011).

In a fourth sham-controlled RCT (Ayache et al., 2016), anodal tDCS was applied for three consecutive days over the left dorsolateral prefrontal cortex (DLPFC), which constitutes a key hub for pain, as well as cognitive and affective networks (Lorenz et al., 2003). The sessions lasted 20 min. In the 16 PwMS with neuropathic pain and predominantly RR disease type, only the active stimulation condition resulted in significant analgesic effects compared to sham [VAS and Brief Pain Inventory (BPI)], without significant changes in the other variables including attention, anxiety, depression, fatigue, and pain-related neurophysiological measures. Interestingly, in this study, tDCS-induced analgesia was observed using VAS and on the pain interference but not on the intensity subscale of BPI. Similarly, in a fifth sham-controlled RCT by the same team, three consecutive daily sessions of anodal tRNS (20 min per session) were applied over the left DLPFC in 16 people suffering from neuropathic pain and predominantly RR MS (Palm et al., 2016). As with the previous study, pain (VAS and BPI), attention, anxiety, depression, fatigue, and pain-related evoked potentials were assessed. Here, there was a trend toward a decrease in pain scores and pain-related evoked potential amplitudes. The application of tRNS is a strength of this study, as it has rarely been used in this clinical population. The lack of statistical significance might be related to some factors, such as the small sample size, which may have been underpowered, the relatively short protocol duration (three consecutive daily sessions may not be sufficient for tRNS effects to emerge), and the concomitant treatments (i.e., antiepileptics and antidepressants).

In a sixth pilot study, the authors assessed the effects of five consecutive daily sessions of anodal tDCS over M1 representation of the most affected leg in six RR PwMS (Workman et al., 2020). They performed a crossover sham-controlled RCT and were interested in investigating the effects of tDCS on MS symptoms that tend to cluster together, namely pain (VAS), fatigue and fatigability, and depression. Here, tDCS significantly decreased pain, fatigability, and fatigue, but not depression. In this study, PwMS had either no or mild depressive symptoms, which may explain the latter finding.

Finally, a sham-controlled RCT compared HF rTMS and iTBS in 34 secondary progressive PwMS suffering from spasticity, fatigue, and spasticity-related pain (measured by a dedicated questionnaire) (Korzhova et al., 2019). Neuronavigation was used to guide the stimulation of M1. Compared to sham, both interventions led to antispastic effects. However, only HF rTMS resulted in analgesic and antifatigue effects that lasted 2 weeks post-intervention, whereas only iTBS resulted in significant long-term antispastic effects.

In all these reports, NIBS sessions were well tolerated, and no serious adverse effects were reported at any time. Two trials included the Comfort Rating Questionnaire and showed no difference in overall comfort and sensations between active and sham conditions (Ayache et al., 2016; Palm et al., 2016). Blinding integrity was assessed in three trials with inconsistent findings. There was no significant difference in guessing the stimulation condition in the two trials (Ayache et al., 2016; Palm et al., 2016), while 56% of individuals could correctly guess the condition in the third trial (Young et al., 2020). This information was not provided in the remaining reports.

## 4 Discussion

Most of the available neuromodulation data for pain management in PwMS consisted of anodal tDCS applied over the precentral area (M1) contralateral to the pain site or the left DLPFC and yielded significant results. This concerned mainly neuropathic pain in PwMS, mostly suffering from RR disease type. In addition, one study applied tRNS and showed a trend toward improving pain perception and pain-related neurophysiological processes. Finally, only one rTMS/iTBS study was available stimulating M1 contralateral to pain site in secondary progressive PwMS, suffering from spasticity-related pain, and the results seem promising.

The observed analgesic effects in PwMS are consistent with the results of a systematic review involving individuals suffering from chronic pain due to various pathologies (Vaseghi et al., 2014). Here, anodal stimulation of either the M1 or the left DLPFC resulted in significant effects, with a larger effect size obtained with the latter setup (Vaseghi et al., 2014). To interpret the mechanisms of action of tDCS/tRNS-induced analgesia according to the site of stimulation, it is interesting to consider the concept of the “pain matrix,” which encompasses three-order networks (Garcia-Larrea and Peyron, 2013): (1) a first-order network that receives spinothalamic projections and contains the posterior operculum and insular regions, (2) a second order network that ensures the transition from cortical nociception to conscious perception and includes prefrontal, posterior parietal, and anterior insular regions, and (3) a third order network that allows the modulation of perception according to emotions, beliefs, and expectations and includes limbic, perigenual, and orbitofrontal regions. Having tackled the pain matrix, the results of Mori et al., Rudroff et al., Workman et al., and Young et al. may have occurred through the top-down modulation of the sensory-discriminative aspect of pain, while the results of Ayache et al. may have addressed the affective-motivational aspect of pain. The differential effects of targeting M1 vs. DLPFC could be illustrated by a sham-controlled crossover RCT study in which 10 healthy individuals received a single session of anodal tDCS at each cortical site, and the change in functional connectivity within cerebral pain networks was assessed (Sankarasubramanian et al., 2017). According to this proof-of-concept small-scale study, targeting M1 might result in a greater increase in the functional connectivity between the sensory (ventroposterolateral) thalamic nucleus and sensorimotor cortex, whereas targeting DLPFC might exclusively increase the functional connectivity between the affective (medial dorsal) thalamic nucleus and cortical regions involved in affective processing. Future research would benefit from comparing the effects of DLPFC vs. M1 tDCS in PwMS and assessing improvement in the sensory vs. affective components of pain using instruments that include separate subscores (e.g., McGill Pain Questionnaire) (Melzack, 1975).

Although the results of the above-mentioned studies are encouraging and support the safety and beneficial analgesic effects of these techniques, several limitations should be highlighted. The trials included 121 PwMS, with sample sizes ranging from 6 to 34. The cohorts were heterogeneous regarding disease phenotype, disability scores, and concomitant treatments. Pain was assessed using different scales, making comparisons between studies difficult. NIBS was applied to DLFC or M1 cortical sites, but no comparative studies are available. The number of NIBS sessions was limited to three–five consecutive days and the protocols included a relatively short follow-up (most assessed the outcomes immediately after the intervention, with some of them up to the fourth week post-intervention). Some limitations are specific to tDCS/tRNS. This is the case of relatively large electrode sizes (25 or 35 cm^2^), which could result in low electric field (EF) focality and current spread to neighboring regions (e.g., to the primary sensory cortex in the case of M1 stimulation). Another limitation is the design of sham conditions (i.e., ramping down the current at the beginning of the session to mimic the cutaneous sensation of the active condition). Although the sham design used in these studies is the most frequent and acceptable one, some authors suggest that such a design might lead to inadequate blinding and induce neurobiological effects (Fonteneau et al., 2019).

Therefore, several points merit consideration. First, regarding the optimization of NIBS protocols, future studies could benefit from comparing different parameters and techniques and optimizing stimulation protocols accordingly. For instance, comparing M1 vs. DLPFC stimulation would help better understand their potentially different effects. Moreover, controlling for covariates of interest in statistical analysis, such as disease phenotype, disability, disease-modifying therapies, medications, and other MS symptoms, would help draw more formal conclusions about the effects of NIBS. In addition, given the dose–response relationship sometimes observed with NIBS, increasing the number of sessions and lengthening the follow-up period may lead to cumulative clinical effects (Hutton et al., 2023). Moreover, with respect to tDCS/tRNS protocols, one way to improve EF focality might be to use small round electrodes, but at the risk of interindividual variability in EF (Mikkonen et al., 2020). Using a high-definition setup (one central electrode and two or more reference electrodes) could help overcome this drawback and improve focality (Mikkonen et al., 2020; Solomons and Shanmugasundaram, 2020). Furthermore, the sham design remains to be optimized in future tDCS/tRNS trials. Nevertheless, although most of the studies tackling pain in MS focused on tDCS, the mechanisms of action of tDCS vs. rTMS do not appear to be similar, even when targeting the same cortical area (Lefaucheur et al., 2020). Therefore, it would be interesting to assess the role of these different techniques in MS pain. Although the only comparative RCT study in neuropathic pain secondary to radiculopathy found significant analgesia after rTMS not tDCS, another study suggests the beneficial effects of anodal M1 tDCS in individuals with neuropathic pain with previous non-response to HF rTMS (Lefaucheur et al., 2017).

Second, the assessment of pain should consider the different aspects of the symptom, including the sensory and affective components, intensity, interference with daily life, and catastrophic pain-related cognitions. Therefore, it would be helpful to adopt a standardized and comprehensive assessment of pain that combines unidimensional (e.g., VAS) and multidimensional scales that could capture the different aspects of this complaint and allow comparisons of results across NIBS studies. For example, BPI provides two subscores for assessing pain intensity and interference (Cleeland and Ryan, 1994). Another tool is the McGill Pain Questionnaire, which generates two subscores that denote the sensory and affective aspects of pain (Melzack, 1975). In addition, the Pain Catastrophizing Scale allows the assessment of catastrophic pain-related thoughts (e.g., magnification, rumination, and helplessness) (Sullivan et al., 1995). It is also important to assess and account for other invisible MS symptoms (fatigue, anxiety, depression, cognitive deficits, alexithymia, and sleep disorders) that may cluster and interact with pain, share common underlying mechanisms, and interfere with treatment response (Workman et al., 2020; Carvalho et al., 2023; Ayache and Chalah, 2024).

Third, one way to optimize treatment response and achieve synergistic or cumulative effects might be to combine several therapeutic approaches, including psychotherapies (Bäckryd et al., 2024), physical therapies (Shkodina et al., 2024), neurofeedback (Ayache et al., 2021), and interoceptive technologies (Schoeller et al., 2024). This approach would ideally facilitate the development of a multimodal, person-tailored treatment plan that considers the type and severity of pain, associated symptoms, and individual preferences.
